# Transfer accuracy and chairside efficiency of rigid- and flexible-printed versus double vacuum-formed lingual indirect bonding trays: a prospective cohort study

**DOI:** 10.1038/s41598-025-14069-x

**Published:** 2025-08-11

**Authors:** Viet Anh Nguyen, Thi Quynh Trang Vuong

**Affiliations:** 1 Faculty of Dentistry, Phenikaa University, Hanoi, Vietnam; 2 Private Practice, Viet Anh Orthodontic Clinic, Hanoi, Vietnam

**Keywords:** Indirect bonding, 3D printing, Lingual brackets, Vacuum-forming, Transfer accuracy, Dental diseases, Oral diseases, Dentistry, Biomedical engineering

## Abstract

This study aimed to compare bracket-transfer accuracy, chairside time, and early bond failure among rigid 3D-printed (RP), flexible 3D-printed (FP), and double vacuum-formed (DV) lingual indirect bonding trays. Thirty-three consecutive adults (*n* = 11) were prospectively enrolled. After virtual setup and tray fabrication, brackets were bonded following a standard protocol. Post-bonding intraoral scans were superimposed on the planned setup, and six positional discrepancies (mesial-distal, in-out, height, rotation, tip, and torque) were quantified for each tooth. One-sample Wilcoxon signed-rank tests compared observed errors with clinical limits (0.5 mm, 2.0°). Kruskal-Wallis and pairwise Mann-Whitney U tests assessed inter-tray differences. 818 brackets (RP = 280; FP = 259; DV = 279) were analysed. Median translational errors were ≤ 0.10 mm for RP/FP and ≤ 0.07 mm for DV; all were below the 0.5 mm threshold (*P* < 0.001). Median pure-rotation errors remained ≤ 1.0° for all trays. Tip met the 2.0° limit except for FP in the lower-anterior segment (*P* = 0.184). Torque rarely met the limit, with RP-upper-anterior being the sole exception. DV outperformed RP and FP in mesial-distal accuracy across upper and lower-posterior regions (*P* ≤ 0.002). All tray systems delivered clinically acceptable translational accuracy, but DV trays provided the most precise mesial-distal positioning. Selecting a tray architecture that balances rigidity and elasticity can markedly improve chairside efficiency without compromising bracket-placement accuracy.

**Supplementary Information:**

The online version contains supplementary material available at 10.1038/s41598-025-14069-x.

## Introduction

Precise bracket placement is integral to efficient orthodontic treatment, yet direct bonding on either the labial or the lingual surface is technique-sensitive, time-consuming, and operator-dependent^1,2^. The advent of digital workflows, including high-resolution intraoral scanning, virtual setup, and computer-aided design and manufacture, has renewed interest in indirect bonding as a means of improving accuracy while shortening chairside time^3,4^. Contemporary indirect bonding trays are produced either by direct three-dimensional (3D) printing (using rigid or flexible photopolymer resins) or by forming on a 3D-printed master model. The latter subgroup encompasses vacuum-formed thermoplastic sheets, auto-cured resin shells, and transparent silicone trays^4–7^.

A growing body of evidence indicates that all three tray types can deliver sub-millimetre linear accuracy and rotation errors close to 1° in labial bonding^8^. Similar findings have been reported for lingual appliances, where a recent in-vivo study of DV trays achieved mean deviations of 0.06–0.12 mm for mesio-distal, in-out, and height, and about 1.3° for rotation^9^. Nevertheless, two consistent patterns emerge from the literature. First, torque is the least accurate dimension regardless of tray design^8,9^. Second, published studies tend to evaluate a single tray material in isolation, whereas direct clinical comparisons between 3D-printed and vacuum-formed systems, especially for lingual appliances, remain scarce^10,11^.

To bridge this gap, the present observational study evaluated three lingual tray systems, including rigid printed (RP), flexible printed (FP), and double vacuum-formed (DV) trays, in the same clinical cohort, measuring bracket-transfer accuracy in six degrees of freedom, recording chairside time, and immediate bond failure. We tested the null hypothesis that the three trays would not differ in accuracy, efficiency, or failure rate. By clarifying these differences, the study aims to guide clinicians toward the most precise and cost‑effective tray choice for lingual indirect bonding.

## Materials and methods

### Ethical approval and study design

The study protocol was reviewed and approved by the Institutional Review Board of Hanoi Medical University (HMUIRB-970) and adhered to the Declaration of Helsinki. Consecutive adult patients who received lingual appliances in a private orthodontic practice (Cau Giay, Hanoi) between July 2023 and July 2024 were consecutively enrolled in an observational cohort study, without randomisation or allocation concealment. Inclusion criteria were: (1) age 18–60 years; (2) complete permanent dentition from second molar to second molar; and (3) absence of morphologic tooth anomalies such as peg laterals or macrodontia. Written informed consent was obtained from all participants. Sample‑size calculation was performed with G*Power 3.1 (Heinrich Heine University Düsseldorf, Düsseldorf, Germany) using a fixed‑effects one‑way ANOVA model, assuming a small effect size (f = 0.25), α = 0.05, and 80% power, a minimum of 159 brackets in total (53 per tray group) was required.

### Digital indirect-bonding workflow

**Fig. 1:**
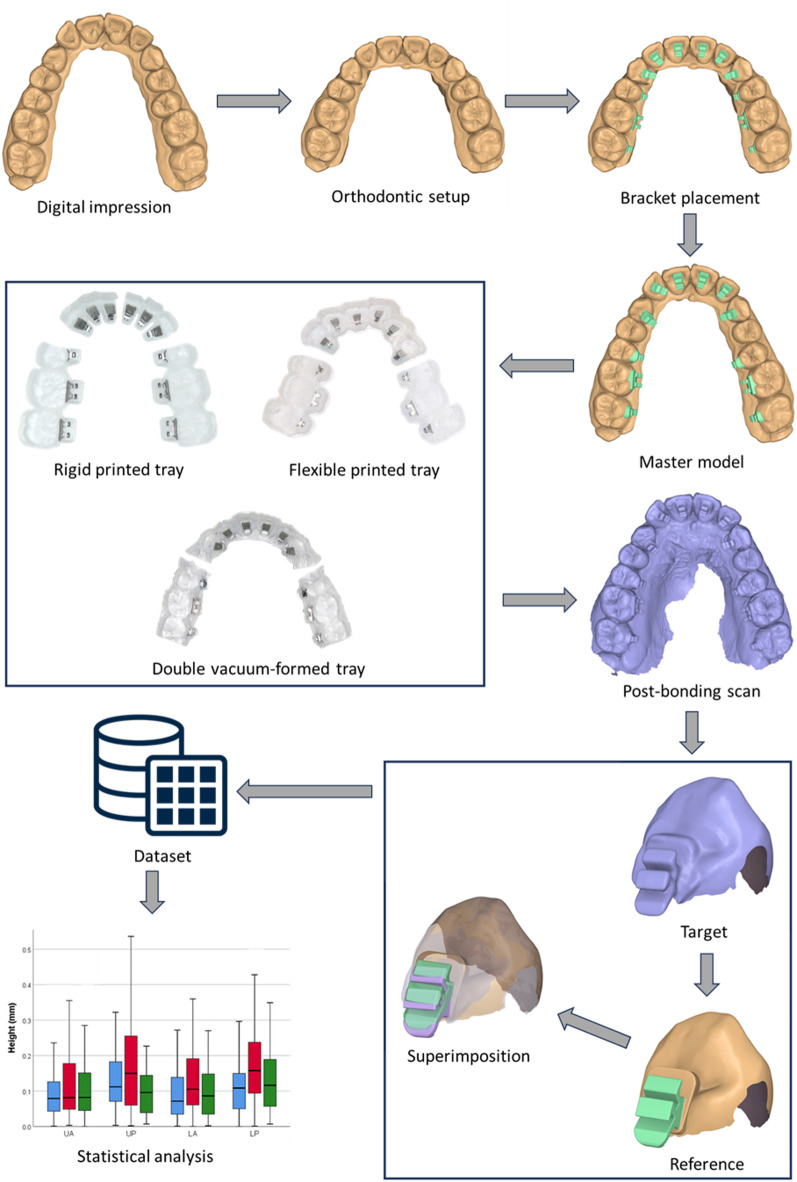
Study workflow.

Intraoral scans of both arches were acquired with an i700 scanner (Medit, Seoul, Korea) and imported into Autolign software (Diorco, Gyeonggi-do, Korea). After automatic segmentation of teeth and gingiva, an ideal setup was generated with full alignment, levelled marginal ridges, and specified tip and torque values. Brackets were virtually positioned on a straight archwire template; their 3D positions were then “locked,” and each tooth was digitally returned to its pretreatment malocclusion^9^. This malocclusion model, with brackets in the planned positions, served both as the master model for tray fabrication and as the reference dataset for subsequent accuracy assessment (Fig. 1).

### Tray groups and fabrication procedures

Each patient was treated with one of three indirect-bonding trays according to clinician preference and material availability (non-random allocation). Rigid 3D-printed trays (RP) were generated by offsetting the bracketed master model by 0.03 mm to form the inner surface and adding a 1.5-mm outer shell for strength; the trays were printed upsidedown (180°) with conventional supports in Surgical Guide Rigid resin (Ludent, Gyeonggi-do, Korea), then cleaned in isopropanol, air-dried and UV-cured according to the manufacturer’s protocol. Flexible 3D-printed trays (FP) used the same 0.03-mm inner offset but a 2.0-mm outer shell to compensate for the lower modulus of the Ortho IBT resin (Ludent). Because this material prints unreliably with supports, the tray base was extruded down to the build platform, and the entire object was printed upside down without additional supports^12^. Post-processing followed manufacturer instructions (isopropanol rinse, nitrogen air-dry, and UV post-cure). All printed trays were designed in Meshmixer v3.5 (Autodesk, San Rafael, CA, USA); a gingival window was left occlusally open around each bracket to avoid unsupported resin during printing.

The double vacuum-formed trays (DV) were fabricated in two pressure-thermoforming steps on the virtually-bracketed master model, with an inner 1-mm Bioplast layer applied for elasticity, followed by a 1-mm Biocryl outer shell to impart rigidity (both Scheu-Dental, Iserlohn, Germany), using a pressure unit (AX-PMU4; IRIS, Tianjin, China).

### Bracket loading and tray stabilisation

Lingual brackets (ADB; Medico, Seoul, Korea) were inserted into each tray. In RP and DV trays, the brackets were stable owing to the tray’s rigidity or full undercut capture, respectively. In FP trays, brackets were reinforced with a light-cured gingival barrier (Top Dam Blue; FGM, Joinville, Brazil) to prevent displacement during seating. In the FP and DV groups, each tray was sectioned into three segments, including two posterior and one anterior. On the other hand, the more rigid RP trays were divided into four shorter segments (two anterior, two posterior) because a long, continuous rigid segment would hamper the path of insertion and removal in the presence of lingual undercuts.

### Clinical bonding protocol

Teeth were prophylactically cleaned and isolated. Enamel was etched for 30 s with 37% phosphoric acid gel (FineEtch; Pulpdent, Watertown, MA, USA), rinsed, and air-dried. A thin coat of primer (Assure Plus, Reliance, Illinois, USA) was applied, followed by a small amount of adhesive (GoTo, Reliance, Illinois, USA) on each bracket base. Trays were seated with firm pressure and polymerised for 40 s per tooth using a high-intensity LED curing unit (LedF, Woodpecker, Guilin, China).

Tray removal differed by group. RP trays, owing to their rigidity, were partially thinned with a high-speed diamond bur (CD-53 F; Mani, Tochigi, Japan) around each bracket before being peeled away. FP trays were gently peeled away after an explorer was carefully inserted between the bracket and the tray to disengage the gingival dam. DV trays were removed in two steps, first splitting off the rigid outer Biocryl shell, then peeling away the elastic inner Bioplast layer.

### Outcome measures

Immediately after each tray was removed, both arches were rescanned with the i700 intraoral scanner and exported as stereolithography (STL) files. Post-bonding scans were imported, together with the master model that contained the planned bracket positions, into Meshmixer v3.5 inspection software (Autodesk, San Rafael, CA, USA). All imported STL files and planned-setup models were relabeled with random alphanumeric identifiers. The investigator conducting the coordinate-based measurements was unaware of group allocation until after the analysis was complete. For every tooth, the crown surface was isolated and used as the registration block for a single-tooth best-fit superimposition to minimize in vivo scan distortion as demonstrated by Jungbauer et al.^13,14^ A tooth-specific Cartesian coordinate system was then generated from the geometry of the bracket slot, where the *x-*axis paralleled the mesial slot edge, the *z-*axis paralleled the labiolingual slot edge, and the *y-*axis was orthogonal to the *xz* plane. Linear discrepancies were recorded along the *x-axis* (mesial-distal), *z-axis* (in-out), and *y-axis* (height). Angular discrepancies were expressed as rotation (yaw about *y*), tip (mesiodistal angulation about *z*), and torque (buccolingual angulation about *x*). Absolute values were used so that positive and negative deviations did not cancel. Any brackets that failed during bonding were excluded from the accuracy analysis, and teeth with severe crowding that precluded proper bracket placement were likewise omitted. To assess reliability, 10% of brackets were remeasured by the same examiner after a two-week interval and by a second examiner on the same day.

### Statistical analysis

All analyses were conducted in SPSS Statistics v26 (IBM Corp., Armonk, NY, USA). Categorical variables (gender, extraction status, Angle class, bond-failure rates) were compared with Pearson χ^2^ tests. Intraclass correlation coefficients (ICC; two-way mixed, absolute agreement) and Bland-Altman analysis were performed to evaluate intra- and interobserver agreement and to detect clinically relevant bias. Continuous data were summarised as mean ± standard deviation and median (interquartile range) after inspection of distributional shape with Shapiro-Wilk tests. Because none of the six positional variables conformed to normality, non-parametric procedures were adopted throughout. Transfer accuracy for each tray-tooth group combination was assessed with one‑sided, one‑sample Wilcoxon signed‑rank tests that compared the observed bracket deviations with the predefined clinical thresholds of 0.5 mm (translational) and 2.0° (rotational). Between-tray differences were assessed with Kruskal-Wallis tests. When the omnibus P value was < 0.05, pairwise Mann-Whitney U tests were carried out and Holm adjustment applied. An α level of 0.05 defined statistical significance. No adjustment was made for multiple outcomes across different positional variables, as each represented a distinct clinical dimension.

## Results

Each tray cohort comprised 11 patients, with 280, 268, and 281 brackets bonded in the RP, FP, and DV groups, respectively (Table 1). Baseline characteristics were broadly comparable among tray types, with no significant differences in age (*P* = 0.858), sex distribution (*P* = 0.333), extraction frequency (*P* = 0.192), Angle classification (*P* = 0.241), or initial crowding (*P* = 0.271, Table 1). By contrast, bonding time varied markedly across groups (Kruskal-Wallis *P* < 0.001) and fell stepwise from RP to FP to DV trays (RP-FP, *P* = 0.002; FP-DV, *P* = 0.002, Holm-adjusted). Bracket bond-failure rates also differed overall (χ^2^ *P* = 0.001), where FP showed more failures than RP (*P* = 0.004, Holm-adjusted) but did not differ from DV (*P* = 0.065), while RP and DV were comparable (*P* = 0.50). After exclusion of failed brackets, the accuracy analysis included 280, 259, and 279 brackets in the RP, FP, and DV cohorts, respectively.

**Table 1:** Baseline demographic and clinical characteristics by tray type.

Variable	RP	FP	DV	*P* value
Age (yr)	28.7 ± 7.1 28 (5.5)	27.4 ± 4.9 28 (7.5)	26.5 ± 3.3 27 (3.5)	0.858
Gender (F/M)	9/2	10/1	11/0	0.333
Extraction (Y/N)	6/5	9/2	5/6	0.192
Angle class (I/II/III)	11/0/0	10/0/1	8/2/1	0.241
Bonding time (min)	68.4 ± 12.7 67 (10.5)	45.0 ± 10.8 44 (8.0)	33.0 ± 4.8 33 (5.0)	< 0.001
Failure rate	0/280 (0%)	9/268 (3.4%)	2/283 (0.7%)	0.001
Crowding (mm)	7.6 ± 3.7 7.4 (4.3)	7.4 ± 4.1 6.1 (4.8)	5.8 ± 4.1 5.7 (4.3)	0.271

Values in each cell are presented as mean ± standard deviation and median (interquartile) for continuous variables, or *n* (percentage) for categorical variables.

*RP* rigid 3D printed tray, *FP* flexible 3D printed tray, *DV* double vacuumformed tray. Age, bonding time, and crowding were compared with the Kruskal–Wallis test; gender, extraction status, angle class, and failure rate were compared with χ^2^ tests. Failure rate = number of debonded brackets divided by total bonded teeth per tray group.

Measurement reliability was excellent (Table 2). Intraobserver consistency was outstanding for every variable, with ICCs approaching unity and negligible systematic bias; the 95% limits of agreement never exceeded ± 0.04 mm for linear dimensions or ± 0.14° for angular dimensions. Interobserver agreement was similarly high, lowest for height at 0.878 but ≥ 0.92 for the remaining parameters, and biases remained clinically trivial, with the widest angular limits (rotation) still confined to < ± 0.8°. Collectively, these metrics indicate that both the linear and angular measurements are repeatable and reproducible for the purposes of bracket-transfer accuracy analysis.

**Table 2:** Intra‑ and inter‑observer reliability for bracket‑position measurements.

Variable	*n*	Intra-observer			Inter-observer		
		ICC	Bias	LoA(95%)	ICC	Bias	LoA(95%)
Mesial-distal (mm)	85	0.938	− 0.006	− 0.032 to 0.021	0.920	− 0.006	− 0.043 to 0.031
In-out (mm)	85	0.986	0.003	− 0.013 to 0.018	0.957	− 0.003	− 0.029 to 0.023
Height (mm)	85	0.991	− 0.003	− 0.010 to 0.005	0.878	− 0.014	− 0.041 to 0.012
Rotation (°)	85	0.992	− 0.007	− 0.095 to 0.081	0.932	0.04	− 0.727 to 0.807
Tip (°)	85	0.997	0.01	− 0.087 to 0.108	0.965	0	− 0.340 to 0.341
Torque (°)	85	0.999	− 0.004	− 0.137 to 0.129	0.992	− 0.04	− 0.399 to 0.319

*ICC* intraclass correlation coefficient (two‑way mixed, absolute agreement), *Bias* mean difference between paired measurements, *LoA* limits of agreement.

**Fig. 2:**
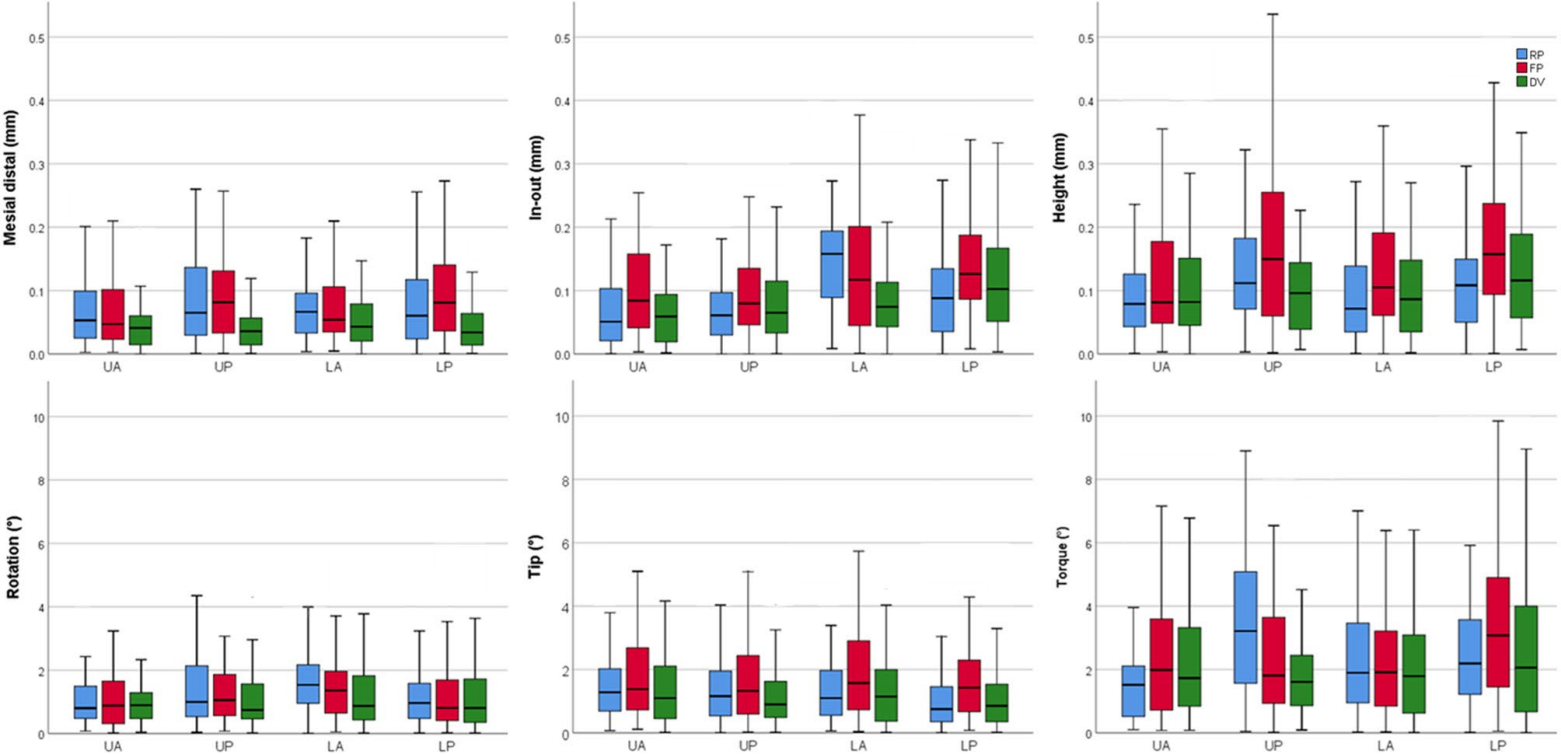
Box-and-whisker plots display the bracket-transfer accuracy across six spatial dimensions, four tooth segments, and three lingual indirect-bonding tray systems.

The positional-deviation data were not normally distributed, hence medians and interquartile ranges are reported in addition to means (Table 3; Fig. 2). Across tooth groups, median translational discrepancies (mesio-distal, in-out, and vertical height) remained ≤ 0.10 mm for RP and FP trays, and ≤ 0.07 mm for DV trays. Median rotational discrepancies were similarly low (≤ 1.00°) for pure rotation, whereas median tip errors ranged from 0.75° to 1.57°. Torque was the most variable parameter, with medians of 1.52°−3.21° for RP, 1.81°−3.07° for FP, and 1.61°−2.06° for DV. One-sided Wilcoxon signed-rank testing (Table II) confirmed that, for every tray type, the medians of all translational variables and pure rotation were significantly below the predefined clinical thresholds (*P* < 0.001 for all comparisons). Tip satisfied the accuracy threshold across nearly all combinations of tray and tooth group, with the only clear exception being the FP tray in the LA segment (*P* = 0.184). In contrast, torque almost never met the threshold, with only the UA segment of the RP trays yielding a median significantly below 2° (*P* < 0.001).

**Table 3:** Descriptive statistics for each tray type and tooth group.

Tray	Tooth group	Mesialdistal	Inout	Height	Rotation	Tip	Torque
RP	UA (65)	0.07 ± 0.06 0.05 (0.07)	0.07 ± 0.08 0.05 (0.08)	0.10 ± 0.09 0.08 (0.08)	1.02 ± 0.82 0.80 (1.02)	1.46 ± 0.99 1.28 (1.34)	1.69 ± 1.59 1.52 (1.59)
	UP (77)	0.09 ± 0.09 0.07 (0.11)	0.07 ± 0.06 0.06 (0.07)	0.13 ± 0.09 0.11 (0.11)	1.41 ± 1.18 1.00 (1.61)	1.39 ± 1.13 1.16 (1.41)	3.49 ± 2.49 3.21 (3.52)^NS^
	LA (62)	0.07 ± 0.05 0.07 (0.06)	0.15 ± 0.08 0.16 (0.10)	0.09 ± 0.07 0.07 (0.11)	1.73 ± 1.17 1.54 (1.22)	1.40 ± 1.10 1.10 (1.42)	2.43 ± 2.00 1.90 (2.52)^NS^
	LP (76)	0.08 ± 0.06 0.06 (0.09)	0.10 ± 0.09 0.09 (0.09)	0.12 ± 0.10 0.11 (0.10)	1.27 ± 1.09 0.96 (1.11)	1.10 ± 1.26 0.75 (1.11)	2.77 ± 2.42 2.19 (2.35)^NS^
	Total (280)	0.08 ± 0.07 0.06 (0.08)	0.10 ± 0.08 0.08 (0.12)	0.11 ± 0.09 0.10 (0.10)	1.35 ± 1.10 1.06 (1.27)	1.33 ± 1.13 1.05 (1.36)	2.64 ± 2.27 1.99 (2.59)^NS^
FP	UA (64)	0.07 ± 0.06 0.05 (0.08)	0.11 ± 0.09 0.08 (0.12)	0.12 ± 0.10 0.08 (0.13)	1.11 ± 0.97 0.89 (1.34)	1.87 ± 1.59 1.38 (1.95)	2.56 ± 2.64 1.98 (2.87)^NS^
	UP (70)	0.09 ± 0.06 0.08 (0.10)	0.10 ± 0.07 0.08 (0.09)	0.19 ± 0.16 0.15 (0.20)	1.31 ± 1.06 1.05 (1.30)	1.79 ± 1.58 1.32 (1.84)	2.37 ± 1.91 1.81 (2.71)^NS^
	LA (53)	0.07 ± 0.05 0.05 (0.07)	0.14 ± 0.10 0.12 (0.16)	0.13 ± 0.09 0.11 (0.13)	1.43 ± 0.92 1.36 (1.32)	1.95 ± 1.54 1.57 (2.17)^NS^	2.17 ± 1.65 1.91 (2.37)^NS^
	LP (72)	0.10 ± 0.09 0.08 (0.10)	0.14 ± 0.08 0.13 (0.10)	0.19 ± 0.17 0.16 (0.14)	1.27 ± 1.31 0.81 (1.28)	1.80 ± 1.65 1.43 (1.63)	3.46 ± 2.62 3.07 (3.45)^NS^
	Total (259)	0.08 ± 0.07 0.06 (0.09)	0.12 ± 0.09 0.10 (0.11)	0.16 ± 0.14 0.13 (0.15)	1.27 ± 1.09 0.98 (1.31)	1.85 ± 1.59 1.42 (1.91)	2.68 ± 2.32 2.25 (2.88)^NS^
DV	UA (63)	0.05 ± 0.04 0.04 (0.05)	0.07 ± 0.08 0.06 (0.08)	0.11 ± 0.10 0.08 (0.11)	1.03 ± 0.83 0.89 (0.83)	1.58 ± 1.47 1.10 (1.70)	2.32 ± 2.01 1.73 (2.62)^NS^
	UP (76)	0.04 ± 0.03 0.04 (0.04)	0.08 ± 0.06 0.07 (0.07)	0.10 ± 0.08 0.10 (0.10)	1.12 ± 0.90 0.74 (1.10)	1.26 ± 1.14 0.90 (1.14)	2.04 ± 1.86 1.61 (1.59)^NS^
	LA (62)	0.05 ± 0.04 0.04 (0.06)	0.09 ± 0.06 0.07 (0.07)	0.11 ± 0.09 0.09 (0.11)	1.13 ± 0.95 0.87 (1.39)	1.31 ± 1.04 1.14 (1.63)	2.03 ± 1.66 1.79 (2.46)^NS^
	LP (78)	0.04 ± 0.04 0.03 (0.05)	0.12 ± 0.09 0.10 (0.12)	0.13 ± 0.10 0.12 (0.13)	1.18 ± 1.12 0.81 (1.37)	1.34 ± 1.57 0.86 (1.18)	2.72 ± 2.71 2.06 (3.33)^NS^
	Total (279)	0.05 ± 0.04 0.04 (0.05)	0.09 ± 0.08 0.07 (0.09)	0.11 ± 0.09 0.09 (0.12)	1.12 ± 0.96 0.84 (1.16)	1.37 ± 1.33 1.00 (1.39)	2.29 ± 2.13 1.74 (2.38)^NS^

Values are shown as mean ± standard deviation (first line) and median (interquartile range) (second line).

*NS* not significantly below the clinical threshold according to one-sided Wilcoxon signed-rank tests, *RP* rigid 3D printed tray, *FP* flexible 3D printed tray, *DV* double vacuum-formed tray, *UA* upper anterior, *UP* upper posterior, *LA* lower anterior, *LP* lower posterior.

Kruskal-Wallis testing revealed only selective, variable-dependent differences among the three tray systems (Table 4). Mesial-distal positioning displayed the clearest advantage for DV, which had lower errors than both RP and FP in UP, LP, and total (*P* < 0.001, Kruskal-Wallis; Holm‑adjusted *p* ≤ 0.002, Mann-Whitney *U*). For the transverse in-out dimension, tray effects appeared in UA (KW = 0.004), where FP showed larger errors than RP (*p* = 0.009) and DV (*p* = 0.010). The contrast reversed in LA (*P* < 0.001, Kruskal-Wallis), with DV outperforming RP (*p* < 0.001) and FP (*p* = 0.039). These differences persisted in the total sample (*P* < 0.001, Kruskal-Wallis; RP-FP, *P* = 0.004; FP-DV, *P* < 0.001). Vertical height errors did not differ among tray types in the anterior segments (UA, *P* = 0.595; LA, *P* = 0.116) but diverged posteriorly (UP, *P* = 0.002; LP, *P* = 0.002). In the UP segment, DV trays produced smaller height deviations than FP trays (*p* = 0.002), with RP trays intermediate. A similar pattern emerged in the LP segment, where FP trays exceeded RP (*p* = 0.002) and DV trays (*p* = 0.032). When all teeth were pooled (*P* < 0.001, Kruskal-Wallis), FP differed from both RP and DV (*p* < 0.001).

**Table 4:** Kruskal-Wallis and pairwise Holm-adjusted *P *values for tray accuracy in each tooth region.

Variable	UA	UP	LA	LP	Total
Mesial-distal	KW:0.048 RP-FP:0.886 RP-DV:0.085 FP-DV:0.079	KW:<0.001 RP-FP:0.802 RP-DV:<0.001 FP-DV:<0.001	KW:0.036 RP-FP:0.842 RP-DV:0.064 FP-DV:0.069	KW:<0.001 RP-FP:0.089 RP-DV:0.002 FP-DV:<0.001	KW:<0.001 RP-FP:0.296 RP-DV:<0.001 FP-DV:<0.001
In-out	KW:0.004 RP-FP:0.009 RP-DV:0.869 FP-DV:0.010	KW:0.090	KW:<0.001 RP-FP:0.165 RP-DV:<0.001 FP-DV:0.039	KW:0.010 RP-FP:0.007 RP-DV:0.224 FP-DV:0.141	KW:<0.001 RP-FP:0.004 RP-DV:0.434 FP-DV:<0.001
Height	KW:0.595	KW:0.002 RP-FP:0.072 RP-DV:0.078 FP-DV:0.002	KW:0.116	KW:0.002 RP-FP:0.002 RP-DV:0.231 FP-DV:0.032	KW:<0.001 RP-FP:<0.001 RP-DV:0.810 FP-DV:<0.001
Rotation	KW:0.973	KW:0.348	KW:0.006 RP-FP:0.196 RP-DV:0.006 FP-DV:0.102	KW:0.632	KW:0.032 RP-FP:0.359 RP-DV:0.029 FP-DV:0.205
Tip	KW:0.372	KW:0.068	KW:0.098	KW:<0.001 RP-FP:<0.001 RP-DV:0.503 FP-DV:0.011	KW:<0.001 RP-FP:<0.001 RP-DV:0.519 FP-DV:<0.001
Torque	KW:0.062	KW:<0.001 RP-FP:0.010 RP-DV:<0.001 FP-DV:0.321	KW:0.605	KW:0.047 RP-FP:0.120 RP-DV:0.577 FP-DV:0.061	KW:0.049 RP-FP:0.720 RP-DV:0.100 FP-DV:0.074

*KW* Kruskal-Wallis omnibus *p*-value comparing rigid 3D-printed trays (RP), flexible 3D-printed trays (FP), and dual vacuum-formed trays (DV) within each tooth region. Post-hoc comparisons (RP-FP, RP-DV, FP-DV) were performed with two-sided Mann-Whitney *U* tests and Holm adjustment for multiplicity. *UA* upperanterior, *UP* upperposterior, *LA* loweranterior, *LP* lowerposterior.

Differences in rotation were limited to LA (*P* = 0.006, Kruskal-Wallis), where DV errors were smaller than RP (*p* = 0.006). This carried over to the pooled analysis (*P* = 0.032, Kruskal-Wallis; DV-RP, *P* = 0.029). For tip, heterogeneity emerged only in LP (*P* < 0.001, Kruskal-Wallis), with FP exhibiting larger tipping errors than RP (*P* < 0.001) and DV (*P* = 0.011). The same hierarchy was evident in the total set (*P* < 0.001 for both comparisons). Torque differences were confined to UP (*P* < 0.001, Kruskal-Wallis), with RP showing greater deviations than FP (*p* = 0.010) and DV (*p* < 0.001).

## Discussion

This study compared the indirect-bonding accuracy of three lingual tray systems, rigid printed, flexible printed, and double vacuum-formed, against clinical thresholds of 0.5 mm and 2.0°. Although all trays delivered translational and purerotation errors that were clinically acceptable, statistically significant differences emerged among systems. DV trays produced the smallest overall discrepancies, particularly in the mesial-distal plane, whereas FP trays showed greater vertical, transverse, and tipping errors, and RP trays displayed a torque deficiency in the upper-posterior segment. These findings partially falsify the null hypothesis of equivalent accuracy across tray types and indicate that material properties and tray architecture materially influence bracket transfer.

Several factors may explain the observed hierarchy. DV trays capture full undercuts around brackets and combine elastic and rigid layers, which enhances seating fidelity yet still allows easy removal. Their comparatively thin profile, with total thickness about 1.2 mm, also facilitates visual confirmation that the tray is fully seated and likely contributes to their superior mesial-distal precision. FP trays rely on a single flexible resin that shortens chairside time but readily deforms under finger pressure during seating. Because the brackets sit loosely, they require stabilisation with a light-cured gingival dam, and the tray’s greater thickness hampers visual control, all of which contribute to increased vertical and in-out scatter. RP trays are dimensionally stable, but because they are printed as continuous rigid shells, they exert greater leverage on the lingual surface during insertion, especially where undercuts are present. Although segmenting the tray mitigated this effect in the anterior region, residual stiffness appears to have compromised torque control in the upper-posterior region, where lingual crowns are more convex.

Our study’s median linear deviations, not exceeding 0.10 mm in every plane and as low as 0.07 mm with the DV tray, mirror the 0.05–0.10 mm mesiodistal band consistently reported in recent meta-analyses of indirect bonding accuracy^5^. Likewise, our buccolingual (in-out) error of ≤ 0.12 mm and vertical error of ≤ 0.13 mm sit comfortably inside the typical 0.08–0.15 mm envelope summarised for contemporary printed and thermoformed guides^9,15–17^. Rotational control was likewise excellent in our sample (medians ≤ 1.0°), matching the ≈ 1° benchmark summarised across multiple systematic reviews and clinical trials^5,9,18^. Tip accuracy (0.75°−1.57°) paralleled the 1.3° mean commonly reported for both printed and vacuum trays, and remained within the 2° clinical threshold across almost all tray-tooth combinations^5,9,17,18^.

Consistent with the broader literature, torque proved the most challenging parameter. Our highest median torque discrepancy (3.21° for RP trays in the UP segment) still fell inside the 2–3° band repeatedly documented as “worstcase” for indirect bonding, whether labial or lingual, printed or vacuum-formed^9,18^. Reviews indicate that barely half of brackets land within 2° for torque, even when linear placement is almost perfect, and that fully enclosing rigid jigs do not consistently overcome this limitation^9,12,18^. Our data, therefore, reinforce the consensus that third-order control, especially on the convex lingual surfaces of molars, remains the last frontier for digital tray systems.

Compared with earlier labial-bonding work, our accuracy hierarchy is inverted. Most labial studies place printed jigs ahead of DV guides. Niu et al.^18^ found smaller mesiodistal errors with a one-piece printed tray designed in 3Shape than with a DV tray, and a recent randomized trial by Mahran et al.^17^ likewise reported superior vertical and buccolingual precision for printed arches, with similar angular control. The difference probably reflects workflow rather than technology. We bonded lingual brackets, which exaggerate undercuts that favour a thin elastic liner, and we modelled trays in generic Meshmixer, whereas those labial studies used dedicated indirect-bonding software that optimises printed-tray geometry.

Within the printed group, our rigid tray still outperformed the flexible version, contradicting the in-vitro and clinical trials of Schwärzler et al.^10,11^ that found no material-related gap. The difference lies in the mechanics because our tray used a stiffer resin, a thicker wall, and was split into four short segments to navigate lingual anatomy, whereas Schwärzler’s “hard” jig was thinner, one-piece, and bonded labial brackets, conditions that let both their “hard” and “soft” resins flex similarly. In a lingual setting, therefore, pairing *true* rigidity with segment-friendly architecture appears essential to unlock the accuracy advantage of printed guides.

Bond-failure patterns paralleled the accuracy findings. RP trays showed the lowest detachment rate, plausibly because the rigid shell was selectively thinned around each bracket with a bur, allowing stress-free tray removal at the cost of extra chairside time. In contrast, neither FP nor DV trays underwent such reduction, where DV partly enveloped bracket undercuts and therefore experienced occasional debonds, while FP recorded the highest failure rate. Several factors may explain FP performance. The tray’s greater thickness hindered visual seating, prolonging manipulation and increasing salivary contamination. Brackets were inherently loose and had to be stabilised with a gingival dam, a step that both lengthened the protocol and risked contaminating the bracket base. Paradoxically, the dam may also have increased retention during tray removal, predisposing to immediate failure.

Chairside efficiency followed the same pattern. DV trays were removed in two quick steps without any bur reduction, so they still yielded the shortest overall bonding time. FP trays demanded more caution, the tray walls were thicker and the brackets were stabilised with a gingival dam, so an explorer had to be teased between bracket and tray to break the dam’s bond before removal, yet the absence of bur work still kept the procedure faster than with RP. RP trays were the slowest because selective tray reduction around every bracket was essential before the shell could be disengaged. Although costs were not formally quantified, the workflow suggests a parallel gradient in economic efficiency. DV trays require only a printed master model and two pressure-forming steps with inexpensive thermoplastic sheets, so material costs and laboratory time are minimal. RP trays involve a single, low-viscosity resin and straightforward post-processing, placing them in the middle of the cost spectrum. FP trays are the most resource-intensive, as the highly viscous flexible resin is relatively expensive and demands meticulous, support-free printing and prolonged post-curing, while bracket stabilisation with gingival dam prolongs laboratory handling. Taken together, DV offers the most cost-effective and time-efficient option, RP provides a balanced compromise, and FP trades speed during printing for higher consumable costs and added clinical steps.

The practical impact of these accuracy gaps is layered. All three tray types delivered median translational errors well below the 0.5 mm threshold and kept pure rotation within 2°, while tip stayed inside 2° in almost every segment. Torque, however, frequently exceeded the 2° benchmark across trays, especially in the posterior regions, so third-order control remains the residual weak point. Although several inter-tray differences reached statistical significance, the absolute magnitudes involved may approach the inherent play between common archwires and 0.018-in bracket slots, typically 0.025–0.051 mm vertically and 8–11° torsion for 0.016 × 0.022-in and 0.017-0.025-in archwires^19,20^. Consequently, routine archwire sequencing, minor compensatory bends or, if necessary, selective bracket repositioning could readily neutralise such discrepancies. However, when precise palatal-root or lingual-crown torque is critical, clinicians may favour the DV tray, which showed the smallest overall torque scatter, or incorporate intentional overcorrections in the virtual setup, especially with a flexible-printed guide. An exception is the upper-anterior region, where the RP tray provided the most accurate torque. Chairside considerations mirror these trade-offs. Clinicians must weigh a slower yet sturdier rigid-printed guide, a faster but slightly less reliable flexible-printed guide, and a rapid, generally accurate DV guide that may become more secure if the inner shell is lightly reducted before removal to minimise early debonds.

Study limitations include the nonrandomised, single-centre design, the use of a single printer-resin combination per tray category, the lack of quantitative assessment of resin flow around the brackets, the decision to bond directly to standard bracket bases rather than custom composite pads, which can consume free methacrylate double bonds during pad polymerisation and thus impair interfacial adhesion with the adhesive. Another limitation is the absence of long-term follow-up to confirm whether initial placement differences affect treatment outcome, because wire-slot clearance can offset small bracket-placement errors. Performance might differ with alternative materials, build orientations, or over time. Future trials should incorporate random allocation, a larger sample, a wider range of resins, quantitative adhesive-flow analysis, evaluation of custom base protocols, and track long-term tooth movement. Notwithstanding these constraints, our data set, covering more than 800 brackets, remains substantially larger than those of previous studies and therefore retains clinical relevance.

## Conclusions

In summary, all three tray systems achieved clinically acceptable indirect-bonding accuracy. Double-vacuum trays delivered the most consistent transfers and the shortest bonding time, flexible-printed trays provided a shorter bonding time than rigid guides but at the cost of modestly lower precision and a higher early-failure rate, and rigid-printed trays remained highly reliable, except for torque in the upper-posterior segment, though they required the longest chairside time. Selecting a tray should therefore balance accuracy requirements, bonding-time constraints, and the clinician’s tolerance for post-bonding adjustments. Because our parallel-group design may be influenced by interpatient variability and small deviations may be masked by wire-slot play, these results should be interpreted with caution.

## Supplementary Information

Below is the link to the electronic supplementary material.

Supplementary Material 1

## Data Availability

All data generated or analyzed during this study are included in this published article and its Supplementary Information file (Dataset.xlsx).
